# Effect of vitamin D insufficiency treatment on fertility outcomes in frozen-thawed embryo transfer cycles: A randomized clinical trial

**Published:** 2014-09

**Authors:** Abbas Aflatoonian, Farideh Arabjahvani, Maryam Eftekhar, Mozhgan Sayadi

**Affiliations:** *Research and Clinical Center for Infertility, Shahid Sadoughi University of Medical Sciences, Yazd, Iran.*

**Keywords:** *Vitamin D*, *Embryo transfer*, *Pregnancy rate*

## Abstract

**Background:** Frozen- thawed embryo transfer is an essential part of ART treatment and outcomes of this procedure are associated with several clinical factors. Several studies have showed an increase level of IVF outcomes in women with sufficient vitamin D.

**Objective:** whether treatment of vitamin D insufficiency can improve pregnancy rates in frozen-thawed embryo transfer cycles.

**Materials and Methods: **This is an interventional, randomized clinical trial. Serum 25-(OH) vitamin D level of 128 women who had undergone IVF/ICSI with cryopreservation of embryos was checked. One hundred fourteen infertile women with insufficient serum vitamin D (less than 30 ng/ml) were included in the study. Fifty seven women were treated with supplementary vitamin D, 50000 IU weekly, for 6-8 weeks and fifty seven women were received no supplementation. One hundred six women completed frozen thawed embryo transfer cycles and included in the final analysis. Primary and secondary outcomes were chemical and clinical pregnancy respectively.

**Results: **Our study did not show any significant difference between vitamin D insufficient and treated women in term of chemical (29.40% vs. 29.10% respectively, p=1.00) or clinical (25.50% vs. 21.80% respectively, p=0.81) pregnancy rates.

**Conclusion:** Vitamin D insufficiency treatment is not associated with higher pregnancy rate in frozen-thawed embryo transfer cycles.

## Introduction

The World Health Organization estimated that approximately 10-25% of couples have infertility disorder. Infertility affects about 80 million people across the world. Assisted reproductive technology (ART) is used to achieve pregnancy by artificial means (1). Frozen thawed embryo transfer has become an essential part of ART (2). Vitamin D is a steroid hormones and acts in the nucleus via binding its nuclear receptors. The main physiologic function of, 1, 25-(OH)2 vitamin D, is to retain calcium homeostasis (3). 

Among the many physiologic functions influenced by vitamin D, important roles in reproductive physiology are suggested. Data regarding the effects of vitamin D on reproductive physiology in non pregnant subjects are limited to a few experimental animal investigations; specific human data are sparse (4). Additional data supporting an association between vitamin D and reproduction comes from investigation of the vitamin D receptors. Vitamin D receptors are seen in different reproductive organs, containing uterus and ovaries (5). The circulating level of the 25-(OH) vitamin D is the universally accepted indicator of vitamin D status (3). In the past, a patient was considered deficient when 25 (OH) D level was <10 ng/ml. Today, controversy exists. Most literature quotes levels <30 ng/ml as insufficiency (6). Vitamin D insufficiency is highly prevalent in reproductive age women and linked to an increased risk for cancer, diabetes, autoimmune and cardiovascular diseases (5, 7). 

More recently, hypovitaminosis D has been implicated as a contributing factor to poor pregnancy outcomes and infertility (8). High prevalence of vitamin D deficiency is seen inIran (9). Prevalence of all stages of vitamin D deficiency is surprisingly high in all cities of iran. Vitamin D deficiency is shown in 72.1% of men and 75.1% of women (10). An IVF population provides valuable insight into the role of vitamin D since it is possible to evaluate each aspect of a single conception cycle from follicular development to implantation (8). IVF Studies investigating the association of vitamin D status with IVF outcome revealed inconsistent results (7). In a study with 84 infertile women undergoing IVF, women with higher levels of 25 (OH) vit D in follicular fluid and serum had significantly higher clinical pregnancy rates following IVF, and high vitamin D levels were significantly associated with better parameters of controlled ovarian hyperstimulation (4). 

In contrast, Aleyasin *et al* could not show a significant association between serum and follicular fluid 25 (OH) vitamin D levels and IVF outcomes in a study including 82 infertile women undergoing ART (11). Anifandis *and co-workers* investigated 101 women undergoing 101 IVF-intracytoplasmatic sperm injection (ICSI) cycles. In their study, women with a sufficient follicular fluid vitamin D status (25 (OH) vitamin D >30 ng/ml) had a poorer quality of embryos and significantly lower clinical pregnancy in comparison with women with deficient vitamin D status (follicular fluid 25 (OH) vitamin D <20 ng/ml) or insufficient (follicular fluid 25 (OH) vitamin D 20.1-30 ng/ml) (12). 

In total, up till now, there is no adequate data to exactly assess the effects of vitamin D in women undergoing IVF. In frozen-thawed embryo transfer cycles we can differentiate factors that impact implantation from factors that influence ovarian stimulation and embryo quality. Thus the effect of vitamin D on the oocytes and endometrium can be distinguished. In addition with treatment of a subgroup of vitamin D insufficient women in frozen-thawed embryo transfer cycles we could evaluate effect of this modality on pregnancy outcomes.

## Materials and methods

We conducted this interventional, randomized clinical trial at Yazd research and clinical for infertility shahid sadoughi university of medical science between September 2013 to April 2014. The study was approved by the Ethics Committee of the Shahid Sadoughi University of Medical Science, Yazd, Iran. Informed written consent was obtained from all couples. Women who had undergone IVF/ICSI with cryopreservation of embryos were entered into the study. 

Third party reproduction cycles were excluded. Women older than 40 years, less than 20 years, those with BMI >30 Kg/m^2^, history of endocrine disorders, severe endometriosis, systemic diseases, repeated implantation failure, repeated abortion, congenital or acquired uterine anomaly with or without operation and also the patients who had visible hydrosalpinx in vaginal ultrasonography were excluded from the study. None of the women had previous history of vitamin D consumption. The patients were allocated into two groups by disclosing the sealed envelopes: vitamin D treated group and control group. The size of samples were defined 50 women in each group based on previous similar studious by statistician. One fasting venous blood sample (5 mL) was taken from each participant. Serum 25-hydroxy vitamin D level was measured with ELISA (enzyme-linked radioimmunosorbent assay) method. We considered patients insufficient or deficient; hear after refer to as insufficient; if they had serum vitamin D below 30ng/ml.

Serum 25-(OH) vitamin D level of 128 women were checked. 14 women had sufficient serum vitamin D (>30 ng/ml). 114 women who their level of serum vitamin D was less than 30 ng/ml were included in this study. 57patients; as a vitamin D group; received 50000 IU vitamin D pearl capsule (50000 IU; Exir Co., Tehran, Iran) weekly, for 6-8 weeks. Serum 25-(OH) vitamin D level was rechecked at least 2 weeks after the end of the treatment. We continued vitamin D 50000 IU monthly for patients who had sufficient serum 25(OH) vitamin D. Another group included of 57 patients; as a control group; were entered in the frozen-thawed embryo transfer cycle without any intervention. 

The endometrial preparation program was similar in both groups. All women received oral estradiol valerate (2 mg, Aburaihan Co., Tehran, Iran) 6 mg daily from the second day of the menstrual cycle. Endometrial thickness was measured by vaginal ultrasonography from the 12^th^ or 13^th^ day of the cycle. The dose was increased to 8 mg in patients with endometrial thickness less than 7 mm. When the endometrial thickness was 7 mm or more, intramuscular (IM) injections of progesterone in oil (100 mg; Aburaihan Co., Tehran, Iran) were administered to the patients. 

Embryo transfer was performed after three days. The cycle was cancelled in the patients with thin endometrium (endometrial thickness <7mm) until 22^nd^ day. We transferred one to three embryos according to age of patient, number and grading of the embryos. Estradiol and progesterone were continued after the embryo transfer till the tenth week of gestational age if pregnancy occurred. Morphological assessment of all embryos was performed on the second day after oocyte retrieval and on the day of frozen thawed embryo transfer. 

Only embryos that had less than 30% fragmentation were frozen by the vitrification. Warming was performed at least three months after cryopreservation. Vitrification and warming was done according to vitrolife instruction. Warmed embryos were transferred to a culture media and evaluated 24 hours later. Embryo evaluation was done. Grade A was considered as equal size blastomeres without any fragmentation. Grade B had slightly unequal blastomere up to 10% fragmentation and Grade C had unequal sized blastomeres up to 50 % fragmentation with large granules. Grade D was considered unequal blastomeres with severe cytoplasmic fragments and large black granules. The grade D embryos were not transferred. 

Primary outcome was chemical pregnancy that was defined by serum β-hCG level >50 IU/L, 14 days after embryo transfer. Secondary outcome was clinical pregnancy that was defined by observation of gestational sac in vaginal ultrasonography, 7-14 days after positive β-hCG.

**Statistical analysis**

Statistical analysis was carried out using SPSS software (Statistical Package for the Social Sciences, version 16.0, SPSS Inc, Chicago, Illinois, USA)Student’s *t* test and chi-square test were used to detect significant differences between two groups. The level of significance was set at p<0.05.

## Results

**Figure 1:**
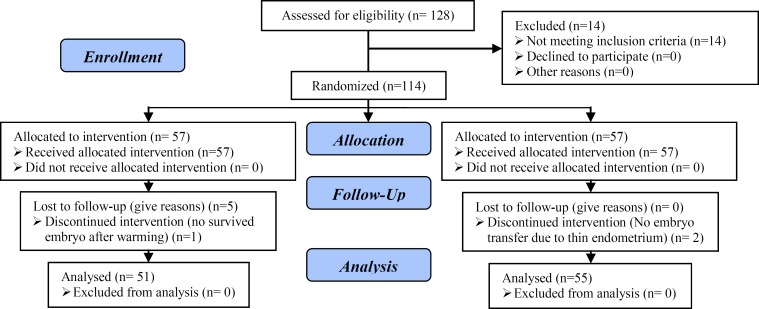
Consort flow chart

In our analysis, we grouped together patients with deficient and insufficient serum vitamin D level as insufficient. We found high prevalence of vitamin D insufficiency in our patients. Only 14 women out of 128 women (10.93%) who checked serum 25-(OH) vitamin D were sufficient. 114 infertile women with insufficient serum vitamin D (less than 30 ng/ml) were included in the study. 57 women were treated with supplementary vitamin D and 57 women received no supplementation. During the study the embryo transfer wasn’t done for two patients in the control group because of having thin endometrium until the 22^nd^ day of the cycle. Additionally, it was not done for one patient in the case group because of no survived embryo after warming. Five patients in the case group gave up the embryo transfer. 106 women completed frozen thawed embryo transfer cycles and included in the final analysis (Figure 1). 

The mean of pretreatment serum 25 (OH) vitamin D in the control group and in the vitamin D group were 15.81 and 14.22 respectively without any significant difference (p=0.18). All patients in vitamin D group had sufficient serum 25 (OH) vitamin D (47.65±1.11) after treatment with vitamin D supplement. There were no statistically significant differences between groups regarding patient demographic and cycle characteristics (Table I, II). We found no significant differences noted in the chemical (p=1.00) and clinical (p=0.81) pregnancy between two groups. (Table III). 

**Table I:** Baseline characteristics in vitamin D treated and control group

**Variables**				**Vitamin D treated group (n=51)**	**Control group (n=55)**	**p-value**
Age (years)				28.45 ± 3.74	29.56 ± 4.68	0.18
BMI (kg/m^2^)				26.87 ± 1.77	26.29 ± 1.67	0.08
Type of infertility						
			Primary	46 (90.20)	49 (89.1)	1.00
			Secondary	5 (9.80)	6 (10.90)	
Etiology of infertility						
			Ovulatory	19 (37.30)	11 (20.00)	0.30
			Tubal	5 (9.80)	4 (7.3 )	
			Male	9 (17.60)	17 (30.90)	
			Mixed	14 (27.50)	18 (32.70)	
			Unexplained	4 (7.8)	4 ( 7.3)	
Type of previous stimulation						
		Antagonist protocol		43 (84.30)	49 (89.10)	0.57
		Agonist protocol		8 (15.70)	6(10.90)	
Cause of embryo freezing						
	Risk of OHSS			22 (43.10)	29 (45.50)	0.84
	Surplus embryos			29 (56.90)	30 (54.5)	
Pretreatment serum vitamin D level, (ng/ml)^x^				15.81 ± 5.94	14.2 ± 6.33	0.18

BMI: body mass index

OHSS: Ovarian hyperstimulation syndrom

Continuous data presented as mean±SD with p-values obtained from Independent- Samples *t* test; Categorical data presented as n (%) with p-value Obtained from Chi-Square test.

x Pretreatment vitamin D: Serum level of 25(OH) vitamin D before treatment

**Table II:** Cycle characteristics of vitamin D treated and control group

**Variables **		**Vitamin D treated group (n=51)**	**Control group (n=55)**	**p-value**
Endometrial thickness, (mm) X		8.93 ± 1.51	8.56 ± 0.86	0.12
Transfer day͓͓ XX		16.29 ± 1.54	15.83 ± 1.11	0.08
No. of Transferred embryos		2.43 ± 0.64	2.54 ± 0.57	0.33
Embryo quality				
	A	15 (29.41)	15 (27.30)	0.70
	B	26 (50.99)	32 (58.20)	
	C	10 (19.60)	8 (14.50)	

Continuous data presented as mean ± SD with p-values obtained from Independent-Samples* t* test; Categorical data presented as N (%) with p-value Obtained from Chi-Square test.

ₓ Endometrial thickness: thickness of endometrium in the first day of progesterone injection.

ₓₓ Transfer day: The frozen- thawed cycle day which the embryos were transferred.

**Table III:** Pregnancy rate in vitamin D treated and control groups

**Variables **	**Vitamin D treated group (n= 51)**	**Control group (n=55)**	**p-value**
Chemical pregnancy rate	15 (29.40)	16 (29.10)	1
Clinical pregnancy rate	13 (25.50)	12 (21.80)	0.81

Data are presented as n (%).

Analysis were performed with the Chi-square test.

## Discussion

Vitamin D deficiency might be important for endocrine disorders including fertility in women. To date, the evidence is based largely on animal and observational studies rather than on intervention trials (7). To our knowledge, this is the first interventional study on infertile women with vitamin D insufficiency in the frozen-thawed embryo transfer cycle. We couldn't show that treatment of serum vitamin D insufficiency increased cycle outcomes in term of chemical or clinical pregnancy. Controversy in the vitamin D is nearly 100 years (7). Most references and literature quotes 30 ng/ml as sufficient level (6). 

The 2011 report from the Institute of Medicine (IOM) recommends a 25 (OH)D level of at least 20 ng/ml (50 nmol/l) based on positive vitamin D effects on bone health (13). Vitamin D deficiency is common in the general population. In many parts of the word 10-60% of adults have values lower than 20ng/ml (14). The mechanism by which vitamin D affects fertility is far from clear. Postulated mechanisms include its effect on ovarian steroidogenesis and implantation (5). Several recent studies explore the role of vitamin D in ART outcomes. Garbedian *et al* assessed the effect of vitamin D status on clinical pregnancy rate following IVF. They found women with sufficient level (>75nmol/l equivalent to >30 ng/ml) of serum 25-(OH) vitamin D have significantly clinical pregnancy, but they didn’t determine embryo quality score, so couldn’t recognize whether endometrial or embryo quality affected implantation and clinical pregnancy rate (5). 

In the ethnically diverse population, Rudick *et al* confirmed that vitamin D status is related to IVF success in non-Hispanic white patients(8). Pregnancy rates declined with progressively lower levels of vitamin D. However, among Asians the beneficial effect of sufficient levels of vitamin D was not seen and indeed, vitamin D status was inversely correlated to IVF success (8). The effect of race on the relationship between vitamin D and clinical pregnancy was statistically significant (8). This study showed that, vitamin D status is not associated with either ovarian stimulation parameters or embryo quality, therefore the effect of vitamin D is likely mediated through the endometrium (8). Rudick *et al* conducted a retrospective study in oocyte donor and recipient cycles and showed that vitamin D insufficiency in the oocyte recipients was associated with lower rates of clinical pregnancy. 

This finding that vitamin D status is associated with IVF outcome among recipients, suggests that the effect of vitamin D is mediated through the endometrium. In this study they could not be analyzed data separately by race (15). Ozkan *et al* assessed 25-(OH) vitamin D levels in the ovarian follicular fluid and determined an association between vitamin D status and cycle outcome. Follicular fluid levels of 25-(OH) vitamin D were significantly higher in women achieving clinical pregnancy following fresh embryo transfer compared with those with failed outcome (4). 

In contrast, Aleyasin *et al* found no significant associations of 25-(OH) D levels in serum and follicular fluid with biochemical or clinical pregnancy rates in a study including 82 infertile women undergoing ART (11). Anifandis *et al* reported adverse impact of higher 25-(OH) D levels on embryo quality. In their study, women with a sufficient follicular fluid vitamin D had a lower quality of embryos and were less likely to achieve clinical pregnancy when compared with women with insufficient or deficient follicular fluid vitamin D (12). 

Altogether, to date, there is insufficient data to accurately evaluate the influence of vitamin D in women undergoing IVF. Contradictory results can be explained not only by methodological differences, but also genetic, racial differences may account to the observed discrepancies in several reproductive outcomes. One important, unresolved issue is the apparent racial heterogeneity in the efficacy of vitamin D. Previous studies have showed significantly lower live birth rates after IVF in Asian ethnicities compared with Caucasians, despite a younger age among Asian subjects and similar embryo quality (8). Numerous studies have shown racial differences in the metabolism of vitamin D. especially; southern Asians have been reported to have increased activity of the enzyme responsible for deactivating both 25-(OH) vit D and calcitriol. There are also ethnic differences in vitamin D receptor (VDR) gene polymorphisms that may confound or modify the relationship between vitamin D levels and reproductive outcomes (8). 

It is possible that the influence of vitamin D on ART outcome is overshadowed by other factors to the lower pregnancy rate observed among Asians and our population. This finding suggests that the relationship between vitamin D and reproduction should be considered within the context of ethnicity. Genetic studies provide excellent opportunities to link molecular insights with epidemiological data. Another explanation for our results is related to the effect of vitamin D in reproductive function. Local production of 1, 25-(OH)_2_ vitamin D by maternal decidua, immune cells, and invading trophoblasts has been proposed to critically regulate implantation and growth at the maternal-fetal surface (16). 

It seems that to be a relation between serum calcium levels and reproductive function (17). VDR null mutant female mice have an underdeveloped uterus and were infertile because of a defect that decreases estrogen production. A high calcium diet maintained 100% fertility in the VDR knockout. It seems that the defect in reproduction is not the lack of a direct influence of 1, 25-dihydroxycholecalciferol on reproductive function but is the result of hypocalcemia (17). Lerchbaum and Obermayer- Pietsch in a systematic review about vitamin D and fertility were emphasized that in infertility patients drastic improvements in reproductive failure may not be achieved by vitamin D treatment alone; however, vitamin D supplementation is a safe and cheap treatment, which might have some beneficial effects on human reproduction (7). 

The prevalence of vitamin D insufficiency was about 90% in our population. This is alarming high and more than the previous reports in the most of the other countries. Shortsun exposure span, nutritional and clothing habits may cause this difference. To date, there is no specific guideline regarding vitamin D supplementation for women affected by endocrine disturbances including infertility. High-quality RCTs with a large sample size are required to determine the optimal 25-(OH) vitamin D levels and to evaluate the effect of vitamin D supplementation on fertility.

## Footnote

***Registration ID in IRCT: IRCT201207156420N11***

***This article extracted from fellowship course thesis. (Farideh Arabjahvani)***
