# Correction to “Effect of vitamin D insufficiency treatment on fertility outcomes in frozen-thawed embryo transfer cycles: A randomized clinical trial” [Iran J Reprod Med 2014; 12: 595-600]

**DOI:** 10.18502/ijrm.v20i1.10410

**Published:** 2022-02-18

**Authors:** Abbas Aflatoonian, Farideh Arabjahvani, Maryam Eftekhar, Mozhgan Sayadi

**Table 1:** (continued)


**Author, yr (ref)**	**Herbal compounds**	**Model/design**	**Duration/doses**	**Results (therapeutic effects on PCOS) or mechanism**
**Ndeingang ** ***et al.*** **, 2019 (42)**	*Phyllanthus muellerianus* (*Euphorbiaceae*)	Rats/letrozole induced PCOS	30 mg/kg, 60 mg/kg, 120 mg/kg for seven or 14 days	1. “Alleviated the reproductive, biochemical, and structural alterations in PCOS rats characterized by the restoration of estrus cyclicity, the reduction of blood glucose levels and oxidative stress, as well as the improvement of the lipid profile and sex hormones” 2. Decreased cystic follicles, LH and T levels, but increased E2 concentration 3. “It was proposed that *P. muellerianus *acts by (a) modulating the pulsatile release of GnRH, LH, and FSH, (b) amplifying the aromatization of androgens into estrogens, and (c) stimulating estrogen production by adipocytes, responsible for the restoration of estrus cyclicity and ovulation induction”
**Shao ** ***et al.*** **, 2019 (9)**	Shaoyao-Gancao Decoction (SGD)	Rats/letrozole induced PCOS	12.5 gr/kg, 25 gr/kg, 50 gr/kg for 14 days	1. “Alleviated hyperandrogenism in PCOS rats as evidenced by reduced serum levels of T and increased E2 and FSH levels” 2. “Reduced the phosphorylation of NF-κB p65 and increased the expression of IκB” 3. “SGD could ameliorate hyperandrogenism in PCOS rats, and the potential mechanism may relate to the NF-κB pathway”
FSH: Follicle stimulating hormone, E2: Estradiol, T: Testosterone, LH: Luteinizing hormone, P4: Progesterone, PRL: Prolactin, AMH: Anti-Müllerian hormone, GnRH: Gonadotropin releasing hormone, LDL: Low density lipoprotein, LDL-C: Low density lipoprotein cholesterol, HDL: High density lipoprotein, HDL-C: High density lipoprotein cholesterol, SHBG: Sex hormone binding globulin, COX1: Cyclooxygenase 1, COX2: Cyclooxygenase 2, BMI: Body mass index, HOMA-IR: Homeostatic model assessment for insulin resistance, FIN-D2D: Finnish national diabetes prevention program, TG: Triglyceride, ALT: Alanine aminotransferase, BUN: Blood urea nitrogen, CK: Creatine kinase, ROS: Reactive oxygen species, P.O.: Per os, CMC: Carboxymethyl cellulose, PCOS: Polycystic ovary syndrome				

The authors have informed the journal that some errors occurred in their published article entitled “Effect of vitamin D insufficiency treatment on fertility outcomes in frozenthawed embryo transfer cycles: A randomized clinical trial” [Iran J Reprod Med 2014; 12: 595-600] in the time of study and the tables, which do not affect the article’s conclusion. The completion date of the investigation was changed from April 2014 to February 2014. Also, the study results were re-analyzed, and some minor modifications were made in the numbers mentioned in the tables without affecting the overall meaning and concept of the study.

The author’s wish to apologize for these errors. The online version of the article has been updated on 30 January 2022 and can be found at http://dx.doi.org/10.18502/ijrm.v12i9.582.

